# Explanation

**Published:** 1879-09

**Authors:** 


EXPLANATION. — The article in the present number of the JOURNAL AND EXAMINER, headed, "On Fracture of Basio Cranii," was sent to us by the secretary of the McLean County Medical Society, with the statement that its publication was requested by a vote of the society. It being a good article, although the name of the author was not appended, we sent it to the printer and immediately wrote to the secretary for the name and residence of the author, but up to the time of going to press nothing further has been heard from him. Hence the article appears as an orphan for the present.

